# Mumps Outbreaks in Vaccinated Populations—Is It Time to Re-assess the Clinical Efficacy of Vaccines?

**DOI:** 10.3389/fimmu.2020.02089

**Published:** 2020-09-18

**Authors:** Anna R. Connell, Jeff Connell, T. Ronan Leahy, Jaythoon Hassan

**Affiliations:** ^1^National Children's Research Centre, Children's Health Ireland, Dublin, Ireland; ^2^National Virus Reference Laboratory, University College Dublin, Dublin, Ireland; ^3^Children's Health Ireland, Dublin, Ireland; ^4^Department of Pediatrics, University of Dublin, Trinity College, Dublin, Ireland

**Keywords:** mumps outbreaks, vaccinated populations, immunity, vaccine efficacy, protection

## Abstract

History illustrates the remarkable public health impact of mass vaccination, by dramatically improving life expectancy and reducing the burden of infectious diseases and co-morbidities worldwide. It has been perceived that if an individual adhered to the MMR vaccine schedule that immunity to mumps virus (MuV) would be lifelong. Recent mumps outbreaks in individuals who had received two doses of the Measles Mumps Rubella (MMR) vaccine has challenged the efficacy of the MMR vaccine. However, clinical symptoms, complications, viral shedding and transmission associated with mumps infection has been shown to be reduced in vaccinated individuals, demonstrating a benefit of this vaccine. Therefore, the question of what constitutes a good mumps vaccine and how its impact is assessed in this modern era remains to be addressed. Epidemiology of the individuals most affected by the outbreaks (predominantly young adults) and variance in the circulating MuV genotype have been well-described alluding to a collection of influences such as vaccine hesitancy, heterogeneous vaccine uptake, primary, and/or secondary vaccine failures. This review aims to discuss in detail the interplay of factors thought to be contributing to the current mumps outbreaks seen in highly vaccinated populations. In addition, how mumps diagnoses has progressed and impacted the understanding of mumps infection since a mumps vaccine was first developed, the limitations of current laboratory tests in confirming protection in vaccinated individuals and how vaccine effectiveness is quantified are also considered. By highlighting knowledge gaps within this area, this state-of-the-art review proposes a change of perspective regarding the impact of a vaccine in a highly vaccinated population from a clinical, diagnostic and public perspective, highlighting a need for a paradigm shift on what is considered vaccine immunity.

## Introduction

### Mumps Virus

MuV is an enveloped, non-segmented, negative-sense, single stranded RNA virus that varies between a spherical and pleiomorphic shape of ~200 nm (85–300 nm) (1, 2). MuV is responsible for an acute viral infection, spread by respiratory droplets (via coughs, sneezes) and urine (3, 4). With an incubation period of 14–25 days, MuV replicates in the nasopharynx and regional lymph nodes, with a secondary viremia occurring late in the incubation period (5, 6). MuV can be detected from saliva up to 7 days prior, and as late as 9 days after clinical onset of parotitis (7).

The MuV genome of seven genes consists of 15,384 nucleotides, and encodes six structural proteins and at least two non-structural proteins; the nucleocapsid protein (NP), V protein (V), phosphoprotein (P), matrix (M) protein, fusion (F) protein, small hydrophobic (SH) protein, hemagglutinin-neuraminidase (HN) protein, and large (L) protein. The role of the I protein is not known (1, 6, 8). The SH gene is the most variable region of the MuV genome; a 2–4% intra-variation and 8–18% inter-variation has been documented (9). This gene is used in molecular phylogeny for genotyping and to identify transmission patterns in populations (6). Despite being serologically monotypic, 12 MuV genotypes (A to L) have been described to date (MuV genotypes E and M are omitted, as the MuV previously assigned to these groups were later re-assigned) (1, 9, 10). The geographic distributions of the MuV genotypes varies worldwide but can co-circulate and thus drive temporal shifts in their distribution. Genotype A was frequently isolated in Europe until the 1990's. Currently genotypes C, D, E, G, and H are prevalent in Europe and the United States of America (USA) whereas genotypes B, F and I are more common in Asian countries (Table 1) (10, 18, 86, 87).

**Table 1:** Comparison of vaccine strain, schedule, and coverage in contrast to the circulating mumps strain and reported cases/year within G20 countries who currently utilize mumps containing vaccines as part of the national vaccination schedule.

**Country**	**Vaccine introduced**	**Vaccines (strains)**	**Vaccination schedule**	**Approximate vaccine coverage (VC)**	**Circulating strains**	**Reported cases/Year**
Argentina	1997 (11, 12)	Present: JL (A) strain. During outbreaks, JL/JL derived vaccines preferred among adolescents and adults (12, 13)	MMR 1: 12 MThs MMR 2: 5–6 years (catch up at 11 years). From December 2018, the government pays for all vaccinations (12, 14–16)	2013: MMR1: 94%; MMR2: 82% 2014: MMR1: 95%; MMR2: 96% 2015: MMR1: 89%; MMR2: 87% 2016: MMR1: 90%; MMR2: 88% 2017: MMR1: 90%; MMR2: 91% (14)	D (2005) (17) K/94-98 (until 2013) (18)	3772: 2013 87: 2014 156: 2015 74: 2016 4396: 2017 771: 2018 (19)
Australia	1982 (20)	JL (A) (21)	1982: MuCV: 12 MThs 1989: MMR 1: 12 MThs 1996: MMR 2: for adolescents 1998: MMR 1: 12 MThs; MMR 2: 4 years. Catch-up between 4 and 16 years. 2013: MMR 1: 12 MThs. MMR 2 (MMRV): 18 MThs (20, 22)	1998: Proof of immunization/exemption required for welfare benefits. 2016: Immunizations required for Family Tax Benefit “No Jab, No Play” 2017: 93% at 2 years. (22, 23)	J/07-08 (until 2013) G (2015) (18, 24)	216: 2013 187: 2014 633: 2015 800: 2016 806: 2017 634: 2018 (19)
Brazil	1992 (25)	1992: Urabe (B) (MMR campaign) 1997: Urabe (B) and Leningrad–Zagreb (N) (MMR campaign) 2003: RIT 4385 (A) (25–29)	2013: MMR 1: 12 MThs; MMR 2: 4–6 years. Booster 1: 11–19 years. Booster 2: After 20 years. 2016: MMR 1: 12 MThs; MMR 2: 15 MThs. Two additional boosters before 20 years, OR a single dose if over 20 years. (25, 26)	2013: MMR1: 100%; MMR2: 69% 2014: MMR1: 100%; MMR2: 89% 2015: MMR1: 96%; MMR2: 80% 2016: MMR1: 95%; MMR2: 77% 2017: MMR1: 97%; MMR2: 41% (14)	K/07(CAN) and K (until 2013) (18, 30)	2014–2015: 82% increase in reported cases in São Paulo (31)
Canada	1969: MuCV 1972: Trivalent MuCV	Mid-1980's (Urabe Am9 MuCV). Withdrawn late 1980's. 1970's: JL (A). Two different MuCVs are used interchangeably (32)	MMR or MMRV vaccine. MMR 1: 12–15 MThs MMR 2: 18 MThs. No later than around school entry (33)	VC of 2 doses of MuCV in school-aged children has been 90% for the past 10 years. in Toronto schools VC 2017–2018: 7 years: 87.4%; 17 years; 95% (34)	A/88, C/85, 88, 11–13 Imported: D/07, 08, 09, 11; F/11–12, G/05–13; H/07, 08, 11–13; K/07, 09, 12–13 (until 2013) G (18, 33)	216: 2013 187: 2014 633: 2015 800: 2016 806: 2017 634: 2018 (19, 35, 36)
China	1990's: (voluntary) 2008: (NIP)	Since 1990: Monovalent MuCV Imported: MuCV JL(A) Domestic: MuCV, mostly S79 strain derived from JL(A) (37)	Pre-2008: MuCV was voluntary and at own expense. 2008-present: MuCV introduced into NIP. One dose of MuCV at 18–24 MThs (38)	Not Available	F/95, 01–12 (11–12/CAN); J/09 (CHN-HK), G/09–11 (CHN-HK); H/11(CHN-HK) (until 2013) (18) 2013–2015: F (99%), G (1%) (38); K (39)	327759: 2013 187500: 2014 182833: 2015 175001: 2016 252740: 2017 259071: 2018 (19)
France	1983	1983: Monovalent MuCV; Urabe (B) 1986: MMR. Urabe (B) 1992: MMR of Urabe (B) discontinued 1992-Present: JL (A) (40–42)	2005: VC documented at 24 MThs MMR 1: 12 MThs. MMR 2: 16–18 MThs (catch up 6–17 years) (43)	2009–2013: MMR 1: ~90.4%. MMR 2: 78.2% MuCV compulsory for children born from January 1 2018 (44, 45)	D/89; C/90 (until 2013) (18)	2: 2015 6: 2016 10: 2017 4: 2018 (19)
Germany	Former German Democratic Republic: No MuCV in NIP. Former West Germany (FWG): 1976: (10, 46)	MuCV: JL (A) RIT4385 (A) L-Zagreb (N) [Reviewed in (10, 47)]	FWG: 1976: MuCV at 12 MThs (voluntary); 1980: MuCV in NIP 1991: 2 MMRs. Dose 2 at ≥5 years 1997: MMR 1: 11–14 MThs 1998: MMR 2: 13 MThs−6 years 2001: MMR 1: 11–14 MThs MMRV: 15–23 MThs. Catch up doses: 2–17 years (10, 46)	2009–2013: MMR 1: ~97%; MMR 2 /MMRV: 93%. (41, 43, 48, 49)	A/87, 90; C/87, 90, 92, 93; D/77; N/87; G/05, 10 (until 2013) (18)	837: 2014 699: 2015 741: 2016 652: 2017 534: 2018 (19)
Italy	1980's (50)	Pre-2001: Urabe (B), Rubini (A) (41) 2001: JL (A), RIT4385 (A), Urabe (B) (51)	1999: MMR offered free to all children in the second year of life 2005–2007: Two-dose schedule as part of NIP 2017: MMR mandatory for children born from 2001. MMR 1: 13–15 MThs; MMR 2: 6 years (52–54)	2013–2017: MMR 1: ~88.6%. MMR 2: 84.2% 2018: 94.1% (55)	Genotype G (56)	808: 2013 821: 2014 675: 2015 782: 2016 829: 2017 47: 2018 (57)
Mexico	1998 (58)	Present: Triple Viral SRP (sarampo, parotidite epidémica e rubeola). JL (A)	1998: Two MuCV introduced 2000: MuCV included to NIP Present: MMR 1: 12 MThs; MMR 2: 6 years (59).	2017: MMR1: 79%; MMR2: 62%2016: MMR1: 97%; MMR2: 98% 2015: MMR1: 100%; MMR2:96% 2014: MMR1: 98%; MMR2: 96% 2013: MMR1: 89%; MMR2: 76% (12, 14, 60)	H (2016) (59)	4142: 2014 3399: 2015 3646: 2016 (19, 59, 61)
Russian Federation	1967 (62)	MuCV used of Russian production, in addition to foreign combination vaccines. Leningrad-3 (Genotype unknown) commonly used (43, 62)	MMR 1: 12 MThs MMR 2: 6 years. (62)	2013–2017: MMR 1 VC: ~98% MMR 2 VC: ~97% (63)	N/53; C/94, 02–04; H/02–04 (until 2013) (18) C and H (Novosibirsk) (64)	282: 2013 267: 2014 190: 2015 1106: 2016 4443: 2017 2027: 2018 (19)
Saudi Arabia	1991	Urabe (B) JL (A) (41, 65)	1991: MMR 1: 12 MThs 1993: MMR provided as a part of EPI. Required for birth certificate 1998–2000: MMR school campaign. 2002–present: a 3-dose schedule Measles-containing vaccine: Nine MThs MMR 1: 12 MThs; MMR 2: 4–6 years. (66–69)	1998–2000 Campaign: 96.4% 2000: School Campaign 96.6% 2006: ~99% of children received MMR vaccine in Keddah. Delays in vaccination have been observed2014: MMR Campaign for children in 1st grade (6/7 years) (67–69)	Not Available	3: 2015 14: 2016 47: 2017 118: 2018 (19)
South Korea	1981	1981–1997: Urabe AM9 (B) 1997–2000: Rubini (A) 2000-present: JL (A)	1980: MuCV introduced 1985: MMR vaccine included in NIP 1997: MMR 1: 12–15 MThs; MMR 2: 4–6 years. 2001: MMR mandatory for school entrance (70)	Two-dose MMR VC more than 95% among pre-school children in Korea (70). Increase in mumps cases attributed to Rubini strain (71, 72)	I/97-01; H/98-01, 07-10, F/07-10 (until 2013) (18), H and I (71)	17022: 2013 1121: 2017 19237: 2018 (19)
Turkey	1970's	MMR (Kizamik Kizamikçik Kabakulak (KKK): JL (A) (73, 74)	1970's-1987: As part of NIP. MMR dose 1: Eight MThs; MMR 2: 15 MThs 1987–1998: MMR 1: Nine MThs 2006–present: MMR 1: 9–12 MThs MMR 2: 6 years (compulsory, free) (73, 74)	MMR used to eliminate Measles and rubella. 2013–2016: VC for MMR 1: ~97% VC for MMR 2: 90.5% (75)	Genotype H (2006–2007 winter season) (76) H/05-07 (until 2013) (18)	597: 2013 457: 2014 322: 2015 544: 2016 419: 2017 464: 2018 (19)
United Kingdom	1988 (77)	1988–1992: Urabe (B) (withdrawn) 1992–1998: JL (A) 1998-present: RIT-4385 (A) (77).	MMR 1: 12–13 MThs MMR 2: From 40 MThs (78)	2013–2017: MMR 1: ~92.6%. MMR 2: 88.6% (75, 79) 2017–2018 (at 5 years): MuCV 1: 94.9%; MuCV 2: 87.2% 2019 (at 5 years): MuCV 1: 94.5%; MuCV 2: 86.4% (80)	B/89, 90; C/75, 80s, 90, 98-00, 04, 06; D/96, 97, 99, 01-04; F/99; G/96-13; H/88, 95-96, 98, 00-04; K/99, 02; J/97, 03-06 (until 2013) (18, 81)	4718: 2013 2958: 2014 1008: 2015 974: 2016 2360: 2017 1398: 2018 (19)
United States	1967	JL (A) (82)	1967: MuCV introduced 1977: MuCV advised for >12 MThs 1989: Second MMR at 4–6 years. Current MMR/MMRV: MMR 1: 12–15 MThs MMR 2: 4–6 years. (82, 83)	2013–2017: VC for ≥1 dose MMR: ~91.9%. (19–35 MThs) 2017–2018: VC for two doses MuCV 3 6/7 years: ~ 94.3%. However, MuCV exemption increased to 2.2% (84, 85)	A/45, 50, 63-91; C/08-10; D/09; G/06-10; K/70s, 07, 08, 10; H/88, 06–10 (up until 2013) (18)	584: 2013 1223: 2014 1308: 2015 6369: 2016 6109: 2017 (19)

*NIP, National immunization program; EPI, Extended Program of Immunization; VC, Vaccine Coverage; MuCV, mumps containing vaccine; MMR1, measles mumps rubella dose 1; MMR2, measles mumps rubella dose 2; JL, Jeryl Lynn (Genotype A)*.

### Development of the Mumps Vaccine

Since 1946 numerous mumps vaccines have been developed worldwide, varying in efficacy and safety profiles but primarily consisting of an attenuated live MuV without an adjuvant (6, 87–89). Currently in Europe and for the majority of the G20 countries who have a mumps vaccine in their immunization schedule (Table 1), the mumps vaccine is included as part of the trivalent measles, mumps rubella (MMR) vaccine, and is primarily administered in two doses (90, 91).

The Jeryl Lynn (JL) vaccine, derived from the genotype A MuV strain was first developed in the USA and has been used extensively in the United Kingdom (UK), Ireland and USA since it was licensed in 1967 (92). Derived from a single clinical sample, and propagated in a chick embryo cell culture, two viral isolates (JL2 and JL5) are present, differing by ~414 nucleotides and 87 amino acid changes (93–95).

The RIT 4385 mumps vaccine, developed from the dominant viral component (JL5) in the JL vaccine strain appears to have comparative safety and efficacy (seroconversion) profiles to the JL vaccine strain (87, 96–98). However, since no controlled clinical trials of efficacy have been published to compare the two doses of the two vaccines, the clinical significance of this observation is not known.

Despite the integration of the MMR vaccine into childhood immunization programs, cyclical outbreaks [defined as two or more cases linked by place and time (96)] of MuV have been documented in several highly vaccinated populations such as Ireland and the United Kingdom (6, 97–103). Between August 2018–and January 2020, 3,736 mumps cases were notified in Ireland, primarily affecting individuals between the ages of 15–24 years. Of the 32% of cases that stated vaccination status, 72% had received two doses of the MMR vaccine (104). An upsurge of mumps cases has also occurred in 47 states of the United States over the last 2 decades, primarily affecting people between 18 and 24 years in close contact/shared settings (105). In Indiana, 76.9% of mumps cases (84.9% of university affiliated and 52% of community cases) had documented evidence of MMR vaccination (106). This results in a significant resource burden for public health departments to control.

**Figure 1:**
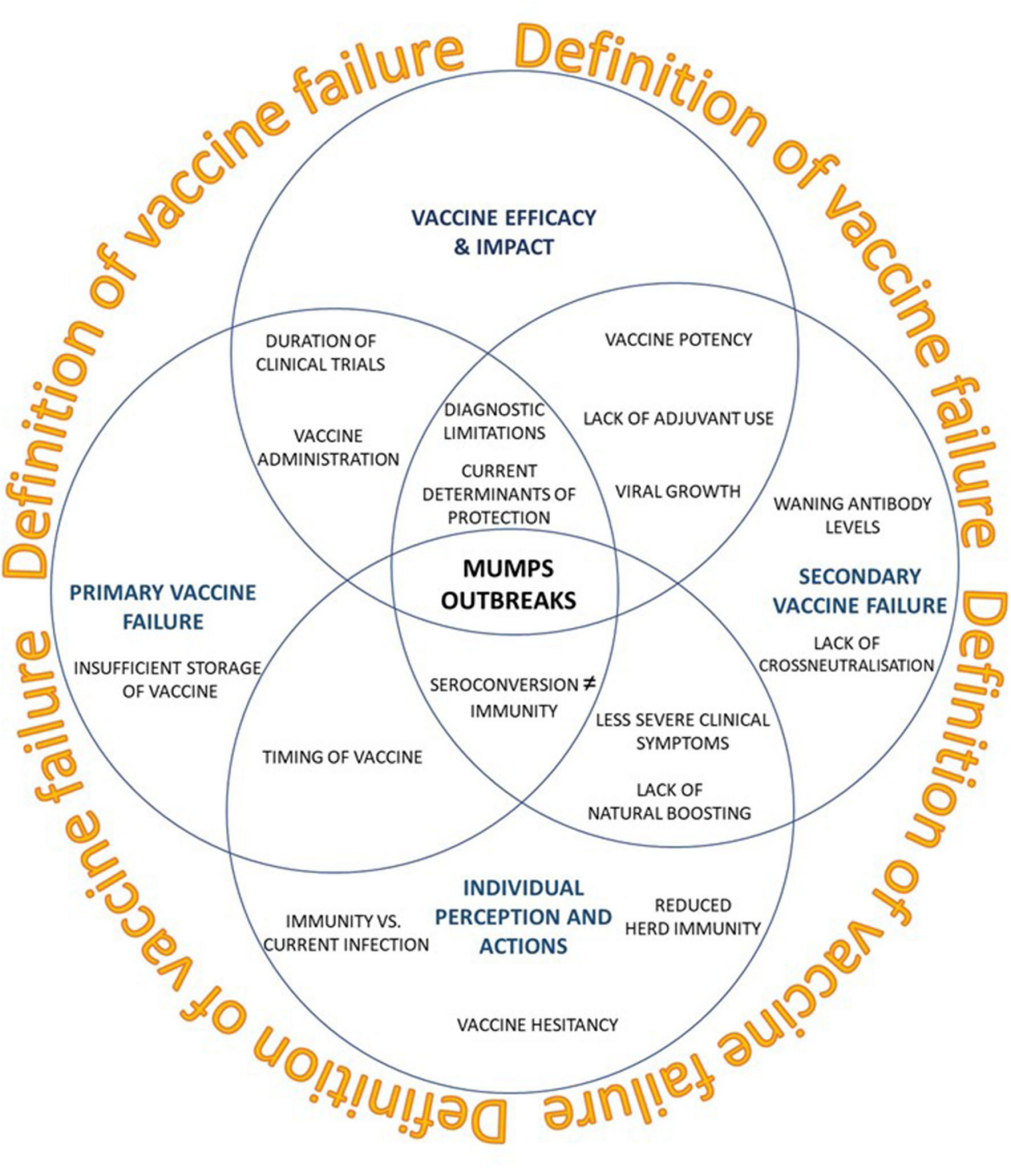
Current perspectives on recent mumps outbreaks seen in vaccinated populations (blue circles). How impactful a vaccine is defined may lead to a paradigm shift in what constitutes an effective vaccine.

Several reviews, both observational and systematic have demonstrated the clinical benefit of a mumps vaccine (107, 108), the pathogenesis and genomic diversity of the MuV (10, 107, 108) and the epidemiology surrounding the outbreak (1, 10, 82). It is not clear why these mumps outbreaks occur, although it has been alluded to be due to a number of interrelated factors, such as sub-optimal vaccine uptake (1, 109, 110), primary or secondary vaccine failure or failure of the mumps vaccine to protect individuals from infection (vaccine efficacy) (107) (Figure 1).

### Vaccine Hesitancy: How Public Perception Predominates

History depicts the remarkable public health impact of mass vaccination. Previously inevitable childhood diseases with potentially debilitating or deadly outcomes have seen their rates plummet worldwide or become successfully eradicated. Immunizations of vaccine preventable diseases are estimated to prevent ~2–3 million deaths per annum and increase life expectancy by ~29 years (111). More recently there has been a shift in the public and media perception of vaccines to their safety, which has facilitated outbreaks such as mumps (112). Organized opposition to vaccinations has a long history; public outcry and resistance following the introduction of the smallpox vaccine in the nineteenth century led to the introduction in England of the Vaccination Act of 1853 (113).

With one in eight children in the USA under the age of 2 currently thought to be unvaccinated due to parental choice, the WHO now considers vaccine hesitancy as one of the ten threats to global health in 2019 (114). Vaccine hesitancy, defined as a “delay in acceptance or refusal of vaccines despite availability of vaccination services” involves a multitude of social, political, cultural and emotional factors in highly vaccinated, western populations (115, 116). One of the main issues is the parental concerns regarding the perceived risk of a vaccine to their child (such as timing/schedules of vaccines, associated pain of administration, and potential adverse effects) vs. the disease morbidity and mortality associated with the vaccine preventable disease (117, 118). The retracted paper published in the Lancet in 1999 (56) and “anti-vaccination” opinions on social media have also contributed to the persistent and insistent misinformation (116), despite vast follow-up epidemiological studies showing no relationship between the MMR vaccine and autism, or differing cognitive development/intelligence (118–120). However, the resultant reaction of the public led to the uptake of the first MMR vaccine falling sharply from 1999, with uptake falling to below 75% in 2002 (104, 121). The age demographic that are experiencing the most cases of mumps in Ireland during the current ongoing outbreak would have been scheduled to have received the first MMR vaccine between 1997 and 2003. Nevertheless, no deductions can be made, due to the lack of vaccination status information provided with reported cases (104).

Heterogeneity of immunization coverage in specific populations or geographic locations of susceptibility is also becoming an important epidemiological issue in maintaining proficient population immunity for mumps (3, 109, 122). The WHO recommends a >95% MMR vaccine coverage for herd immunity. Maintenance of such coverage is well-demonstrated in Finland, where a country-wide 2-dose MMR vaccination program initiated in the 1980's eliminated measles, mumps and rubella within 25 years (123, 124). Recent publications from around the world indicate that the level of MMR vaccine uptake is far lower than what is recommended [reviewed in Ramanathan et al. (125)] (101, 126–129). Of the G20 nations that implement a mumps vaccine within their vaccination schedule, only 3 countries have maintained vaccine coverage levels of >95% (Table 1). However, poor uptake/incomplete vaccination alone may not be the only issue relating to mumps outbreaks. In the Netherlands, mumps outbreaks still occurred with an overall herd immunity threshold of 86–92%, and where 96 and 93% received the first and second MMR at 14 months and 9 years, respectively (125, 130).

## Factors Facilitating Current Mumps Outbreaks in Highly Vaccinated Populations

### The Changing Criteria of Mumps Diagnosis

The clinical presentation of mumps is pathognomic (bi-lateral parotitis); therefore supporting laboratory diagnosis was rarely employed in the past. As the classical symptoms of mumps are not always typical, there may have been a significant number of individuals in the past who may have been infected but were not identified as such. When mumps vaccination was introduced in 1967, the criteria the vaccine had to meet was the proof that it was clinically effective, i.e., that it reduced the risk of disease in vaccinated individuals in real-world conditions over a set period. Such an example was seen the USA; the reported cases (i.e., diagnosis of clinical symptoms) of mumps declined from >100 cases per 100,000 population before 1967 (pre-vaccine era) to 10 cases per 100,000 population in 1977, a reduction of 99% (105, 110, 126, 131). To note, clinical efficacy was probably based upon the reduction of the “classical bilateral presentation” rather than the milder mumps presentation. Therefore, one could argue that the original vaccine efficacy for clinical manifestations was over estimated.

Currently the laboratory diagnosis of mumps infection in Ireland is based upon two approaches: detection of mumps RNA by reverse transcriptase PCR (RT-PCR) in a buccal swab containing saliva, throat swab or urine specimen, and serological detection of immunoglobulin M (IgM) using a capture assay (132, 133). Both approaches for diagnosis are impacted significantly by the quality and timing of sample collection post-onset of symptoms and also if the subject is mumps naïve or had received mumps containing vaccine (87, 126, 134, 135).

There are challenges in using standard serological laboratory diagnostic methods to reliably confirm mumps re-infection of individuals who had been previously naturally infected or vaccinated (130, 136). Briefly, vaccinated individuals re-infected with MuV may only generate a weak or undetectable IgM response (133). Although a rise in IgG titer may also not occur in vaccinated individuals (87, 137), numerous studies have documented a rapid, variable increase in mumps-specific IgG levels, with neutralization antibody concentrations present up to 10 months post-infection (130, 138, 139).

Therefore, Reverse Transcriptase-Polymerase Chain Reaction (RT-PCR) is recommended (133, 140), and was formally introduced in 2015 as the principle diagnostic tool in Ireland to detect mumps in oral fluids (141). RT-PCR can identify current mumps infection more effectively in vaccinated individuals than serological techniques alone as it identifies the presence of the MuV vs. the immunological response (IgG, IgM), and has been previously shown to 100% correlate with viral culture results (140, 141).

The case numbers of more recent mumps outbreaks should always be assessed with this question in mind; are the number of mumps cases increasing, or/and are we better at diagnosing an acute infection? The latter seems to be the most probable, as many individuals who are being tested do not present with classical symptoms. In addition to enhanced surveillance of mumps cases, further optimizations of technologies are also occurring; the utilization of next-generation sequencing demonstrated that by editing one 2-fold degenerate nucleotide in the forward primer and three 2-fold degenerate nucleotides in the probe sequence optimized the fluorescence intensity and clinical sensitivity of the real-time RT-PCR when compared to the CDC-developed and WHO-recommended RT-PCR target [(NP) gene] leading to ~11% increase in clinical sensitivity (i.e., Ct values that were ~3.7 cycles lower) (142).

### Are Primary and Secondary Vaccine Failures Implicated?

Much is not known about the immunological response to the mumps vaccine strain. However, a number of young adults who were vaccinated as children over the last two decades have demonstrated an increased risk of MuV infection with time, which is assumed to be related to a decline of antibodies to sub-protective levels of immunity (40, 101, 125, 128, 143–146).

### Primary Vaccine Failure

Primary vaccine failure is defined as the lack of a sufficient initial antibody response to a vaccine in a recipient resulting in a lack of protective immune responses (6, 147). Although this type of vaccine failure may be because of improper storage/handling or administration of the vaccine, impacting its efficacy, it may also be due to the initial immunological response of an individual to the vaccine, which is usually quantified by the presence of antibodies that should be detectable in the weeks following vaccination. Primary vaccine failure was attributed to primary-school outbreaks of both mumps and measles in Ireland, which subsequently resulted in reducing the age for the second dose of MMR2 vaccine from 10–14 years in 1999 to 4–5 years of age (6). With the cyclical outbreaks occurring, it has been proposed that primary vaccine failure could again be a factor.

How is a response to a vaccine determined? In pre-licensure studies of the JL and Urabe mumps vaccines, high seroconversion and low failure rates were observed in children after the first vaccine dose (>90 and 5.5%, respectively), demonstrating that the vaccine induced an antibody response (148–153). A more recent study by Ong et al. demonstrated that a ≥2-fold increase in mumps antibodies 30-days post-vaccination was considered to be an adequate response of immunity (154). Vaccine effectiveness (i.e., seroconversion post-vaccination) of 2 vaccine doses has only been conducted on the JL strain; 6 studies provided a median vaccine efficacy of 88%. These studies have shown that 2 doses of MMR were more effective (but not statistically significant) than a single MMR dose to combat the incidence of mumps infection (101, 126, 145, 151, 152, 155). Mumps-specific antibodies have been detected 1–2 years post-vaccination and without substantial decline for 8 years after mumps vaccination, with the immunogenicity and efficacy of the MMR vaccine showing comparable immunogenicity levels to post-vaccination levels at 3 years (148, 156). However, most studies of this vaccine (involving either a mumps-specific vaccine or a combined vaccine) only followed-up to 30–56 days post-vaccination (157–167). Despite few follow-up studies estimating post-vaccination antibody titers specific to the vaccine mumps strain, the evidence of seroconversion post-vaccination in a number of studies indicate that primary vaccine failure does not seem to be a significant contributor to the outbreaks that have been recently observed (118, 149, 150, 152, 158, 168–171).

It has been noted that a small percentage of the population do not seroconvert post-vaccination; <1% who received the MMR vaccine were seronegative 4–9 years after the first dose of MMR (*n* = 616) (143). Poor immune responses to primary vaccination has been shown to be a good indicator of infection susceptibility (172). This is in agreement with the correlation of pre-outbreak JL virus neutralization titres and ELISA results being significantly lower in individuals who became infected compared to non-infected individuals (173). Further studies of these individuals may provide insights of which immunological process are integral to develop immunity.

### Secondary Vaccine Failure

The current methods used to determine immunity against mumps cannot discriminate between primary and secondary vaccine failure; only the timing of these tests can assess whether an individual ever mounted an immune response post-vaccination or whether the response is detectable years post-vaccination. Primary vaccine failure encompasses the failure to mount an immune response to a dose of a vaccine, secondary vaccine failure refers to a more gradual loss of immunity after a successful initial response that occurs over a number of years post-vaccination (174). Several factors have been proposed to be implicated with secondary vaccine failure, such as waning immunity, a lack of cross-neutralization, and natural boosting.

### Waning Immunity

Waning immunity is defined as a decline in immunological protection proportional to time since vaccination. Potential waning immunity has been documented in the current mumps outbreaks seen in Europe and the USA, mostly affecting young adults within highly vaccinated populations attending tertiary education who have received two doses of the MMR vaccine in early childhood (40, 110, 126, 144, 145, 175–181).

A number of studies from the USA, where a JL vaccine has been used since 1971 have demonstrated waning immunity within the population. The risk of developing clinical mumps was shown to increase by 10–27% for every year post-MMR vaccination (125), with the rates of mumps infection rising from 1.6 cases per 1,000 in those who received the second dose of the vaccine within 2 years of the outbreak, to 11.3 cases per 1,000 in those who received it over 13 years prior. Using a mathematical model with analytical limitations, a recent meta-analysis of six studies estimated that vaccine-derived immune protection to MuV wanes about 27 years post-vaccination (182). Kennedy et al. (183) also demonstrated a decrease of ~20% in mumps neutralizing antibody titers over 10 years.

In contrast, other studies appear to contradict, these findings, showing no link between mumps protection and time elapsed following administration of mumps vaccine (138, 148, 149, 184, 185). LeBaron et al. (143) and Gothefors et al. (186) demonstrated that 70–99% of individuals still had detectable anti-mumps antibodies ~10 years after initial vaccination. Cohen et al. (101) also demonstrated minimal antibody level decline after two MMR doses 6–7 years after second vaccination. Neutralizing antibodies against the JL-5 vaccine strain has also been detected in ~80% for age groups 2–20 years, 67% for age group 24–26 years; and 77% for age group 50+ years (187).

Implementation of a third dose of the MMR vaccine has been shown to be effective as a stop gap measure in limiting disease spread in outbreak settings situations (129). Individuals vaccinated for the third time had a 78% lower risk of contracting mumps, with a decreased attack rate of 6.7 vs. 14.5 cases per 1,000 when compared to those who received a second dose. More than 50% of those who received a third dose of the MMR vaccine showed a 4-fold increase in mumps antibody titers (105, 106, 168, 188). An increase in mumps IgG humoral immunity was also observed post-vaccine administration. However, this immunity boost has been shown to be a transient effect, with mumps antibody titers returning to pre-third dose of mumps-vaccination levels 1 year after vaccination.

Therefore, as waning immunity is thought to be an important factor facilitating mumps outbreaks, the emphasis placed on the quantity/quality of mumps-specific antibodies may need to be re-assessed. It is yet undetermined if the total loss of detectable antibodies correlates to a loss of clinical protection, as the minimal level of neutralizing antibody required for protection against mumps has not yet been defined (184).

### Cross-Neutralization

Antigenic variation and thus reduced cross-neutralization between the vaccine and circulating strains of different MuV genotypes have been cited as possible explanations for mumps outbreaks in highly vaccinated populations (125, 184, 189–191). Recent outbreaks in Europe and Northern America (including Ireland) have shown the circulating MuV during the current outbreaks to be genotype G (135, 184, 192, 193). This MuV genotype was first identified in 1996, and has demonstrated intra-genotype diversity of up to 7% (Table 1) (6, 134).

The JL vaccine strain (genotype A), differs phylogenetically to the circulating MuV (genotype G) (125). *In vitro* studies of the genotypic distribution and temporal shift of MuV suggest that cross neutralization between wild type and vaccine genotypes may be approximately half the concentration measured against the vaccine strain (130). Pre-infection neutralization titers in mumps positive cases were also significantly lower against genotype G vs. mumps vaccine strain, potentially due to amino acid differences in B-cell epitopes and/or N-linked glycosylation sites on the HN and also within the F protein (194). Santak et al. (195, 196) also demonstrated that conformational changes within the F protein may lead to immunological escape.

Despite the decline/scarcity of cross-neutralizing antibodies, different mumps vaccines used worldwide have been shown to prevent significant clinical mumps infection during outbreaks (101, 197). Dependent on the strain, a 2–16-fold variation of patient sample titers has been shown to be protective in *in vitro* plaque reduction neutralizations (149, 151, 198). Although the sera of one of these studies, was collected only 6 weeks after MMR vaccination, a time point that may not signify the concept of waning immunity and antigenic differences, several other groups have shown that the most divergent strains of MuV can be neutralized *in vitro* with only slight variations in titers, supporting the concept that MuV is serotypically monotypic (184, 190, 195, 198). Epitopes of the MuV that are presented to CD8+ T-cells have been shown to be present in not only the circulating strains of virus but also in a number of vaccine strains (199). In addition, Lewnard et al. (182) also found no evidence that recent mumps outbreaks were due to the emergence of MuV strains escaping vaccine-driven immunological pressure.

Therefore, the limited data does not suggest that antigenic drift of the MuV leading to diminished neutralization capacity of the vaccine strain could fully explain the recent outbreaks (125). Further studies into the cross-neutralizing capacity of the mumps vaccine strain administered 15–20 years previously to the current circulating strain of MuV in countries where outbreaks are being observed will allow better deductions to be made. It is possible that differences in the neutralization capacity of vaccine-induced antibodies against different MuV strains may be more significant when levels of neutralizing antibody are low and become “overwhelmed” when the mumps viral load challenge is high (200).

### Natural Boosting

Several prominent MMR/mumps vaccine studies were undertaken at a time when there was still a high prevalence of circulating wild type virus, which enabled sub-clinical boosting to occur in an individual. Such natural boosting is illustrated in Belarus, where a subpopulation of vaccinated individuals only had a small amount of their overall mumps IgG antibody levels specific to the vaccine-strain (201). Neutralization antibodies against Iowa-G/USA06 (the circulating wild type virus) were also present in pre-infection plasma of all mumps cases during a recent outbreak in the US (173). This indicates that the mumps vaccine alone is not solely responsible for the high levels of mumps antibodies (202), and that long-term antibody persistence or protective efficacy data of the vaccines used may not truly reflect the current circumstance of viral transmission/circulating within a highly vaccinated population (99).

Herd immunity increases the chance for natural mumps boosting for an individual is at a minimum, reducing the potential of the frequency of mumps outbreaks (123, 124, 184). With less opportunity for subclinical boosting (asymptomatic response to the circulating virus), the impact of other elements of waning immunity may play an increasingly critical role in the re-emergence of mumps outbreaks (98, 171). Additionally, as the heterogeneous uptake of vaccines in this modern era is leading to susceptible individuals within the community, future work will need to encompass genotyping of circulating MuV to examine how impactful subclinical boosting was on early measures of vaccine efficacy in current populations.

## Laboratory Determinants of an Effective Immune Response to Mumps Vaccine

### Why Do We Consider Antibodies to Be the Best Measurement of Vaccine Efficacy?

The evolution of an individual's immune response differs between natural infection and vaccination, in particular the difference in the affinity and specificity of an immunological marker such as antibodies (203).

True correlates of mumps immunity after vaccination have been poorly characterized; to date, there are no reliable correlates of protection from either symptomatic mumps infection (clinical immunity), or individuals previously exposed to MuV (204). Therefore, a serological surrogate/ substitute is used (205). Mumps vaccine efficacy is quantified by a single measure, IgG which may not suffice to evaluate the magnitude of the actual humoral response. Borgmann et al. (206) proposed an increase in mumps-specific IgG titer in sera as a diagnostic criteria of mumps reinfection (206). It has been suggested that vaccinated individuals have modified B-cell responses to MuV that allow for the rapid generation of IgG antibodies and a blunted or absent IgM response (207, 208). In addition, emerging data in Simian Immunodeficiency Virus studies suggests that not all antibody responses are equal, and qualitative features of antibodies may be key to defining protective immune profiles (209).

Despite its use, the correlation to mumps-specific IgG concentrations and neutralization titers against the JL virus is poor, suggesting that IgG concentrations do not adequately represent a sufficient surrogate correlate of protection (194). This is demonstrated in Finland; only 24% of vaccinees had no detectable mumps antibodies after 21 years (123, 124). Data from the European Sero-Epidemiology Network (ESEN2) project in 2004 reported that MMR immunization uptake in Ireland in 2004 was 92% (6), however it was also suggested that only 80–85% of 15- to 24-year-olds in Ireland had detectable antibodies to MuV by either natural immunity or immunization (210). In 2011, vaccine coverage of medical students in Germany was reported to be 75.1% (211). In children between the ages of 1–17 years, where 88.8% had been vaccinated with the MMR vaccine at least once, only 76.8% showed prevalence of antibodies (212). However, 7.8% showed a prevalence of antibodies to measles and rubella in the absence of mumps-specific antibodies. Therefore, previous measurement of anti-mumps-specific IgG that represented immunity induced by the mumps vaccine appears to be overestimated (99, 213).

Antibody levels of other components of the MMR vaccine have seen similar trends. Waning rubella antibody titers have been observed, despite the number of acute rubella and congenital rubella syndrome cases not increasing. It has also been shown that college students who received rubella vaccination during childhood and had low/no antibody response were able to mount a secondary response when challenged with rubella indicating that an individual's low antibody levels are not always indicative of susceptibility to infection (214). Measles antibodies can also be detected for up to a decade post-vaccination, with >90% of individuals still measles IgG positive at 6–7 years of age (144, 215). However, as with mumps and rubella, waning measles antibody titers have been observed (143, 216). Despite this, a recent longitudinal study of up to 10 years demonstrates how effective the MMR vaccine has been in preventing diagnosed measles cases during the 1990's/2000's (217).

Similarly, three doses of the Hepatitis B (HBV) vaccine in a cohort of Alaskan natives showed >95% seroconversion in children and young adult post-vaccination and provided long term and durable protection against chronic HBV infection. Although no increase of HBV prevalence were observed 51% individuals had low to undetectable antibody levels after 30 years.

These observations suggest that an individual's antibody levels do not indicate susceptibility to infection, that either an antibody titer lower than recommended guidelines is still protective, or/and is an ineffective surrogate of protection. This is emphasized in a study by Amanna et al.; (218) responses to non-replicating protein antigens (tetanus and diphtheria) were shown to have approximate antibody half-lives of 11–19 years. In comparison, antibodies following wild type infection were shown to have half-lives of 50 years or more which was thought until recently to confer a more prolonged lifelong protection (214, 218, 219). However, reinfections observed in individuals that were previously naturally infected have demonstrated that the quantitative measurement of antibodies do not indicate sterile immunity (220).

It is also important to stress that seroconversion rates due to immunization/natural infection only reflects a change of antibody status from negative to positive, but not necessarily the intensity of antibody response. In addition, there is no consistency in the timing of sample collected post-vaccination to test vaccine efficacy, and between the serological tests utilized for detecting mumps antibodies. As a result, documented seroconversion rates of the mumps vaccines used vary widely (JL: 74–100%, RIT 4385 strain: 88–98%, Urabe Am 9: 79–100%, Rubini: 35–95%).

This highlights that the assays used to detect immunity to MuV may not always detect an adequate post-vaccination response. Only a small number of serological commercial assays such as the detection of Hepatitis B surface antibody (anti-HBs) (221) and rubella IgG (222) have been designed using WHO reference material as a standard for quantification. However, even utilizing this reference standard demonstrates significant differences in the determined quantification of either anti-HBs or rubella IgG depending on the assays used; although a value for anti-HBs of 10 IU/ml is regarded as protective against significant HBV infection, the detection of this anti-HBs is significantly influenced by which anti-HBs assays is used (223–227). Therefore, it is possible that the current assays/tests mechanisms utilized to measure mumps antibodies are too insensitive/inappropriate/crude to identify nuances in the immune response which could correlate with immunity against mumps. In addition, variation within neutralization epitopes i.e., the quality of the antibody present could be a more important correlate than quantity (190, 198).

### Are There Better Correlates of Protection?

Though labor-intensive, neutralizing antibodies are considered to be a better correlate of mumps immunity. Antibodies against the haemagglutinin-neuraminidase protein (HN) and nucleoprotein (NP) have been shown to neutralize MuV, however, repeated attempts to define a titer that provides a protective threshold titer have been inconclusive (203, 228). In older studies, during field evaluations of the JL vaccine, neutralizing antibody titers of 1:2–1:4 in unvaccinated individuals was considered seropositive and protective from mumps infection (149, 151, 152). Using a more contemporary wild-type isolate (Iowa-G/USA06), a 1:8 neutralizing titer cut off was defined between case patients and exposed patients, despite the fact that no cut-off could fully discern between the two groups (173). However, that these results are dependent on the challenge virus strain used in the assay. Rasheed et al. demonstrated a 6-fold lower neutralization titer to the G-genotype when compared to the JL vaccine strain in 18–23 year olds (229). This has also been seen between mumps vaccine strains vs. circulating strains in India and China (47, 197). Despite studies in more highly vaccinated populations demonstrating that HN-inhibiting titers after natural disease were 1:9 compared to 1:5 post-vaccination, neither appeared to prevent reinfection (173, 218–220, 230). There is increasing evidence that the mumps-specific antibody response is broader than neutralization alone (112). Avidity testing for virus-specific IgG has been proposed (3, 220, 229).

### Is Lymphoproliferative Immunity a Better Correlate of Protection?

Individuals who lack measurable mumps-specific antibody levels may be susceptible to infection but protected from significant illness as they may be protected by cell-mediated immune memory. Prolonged T-cell responses are reported after other vaccinations; 14–16 years after a single dose of the rubella vaccine RA27/3, a T-cell proliferative response to neutralizing antibody-inducing peptides suggest T helper and B-cell interactions. This indicates that full vaccine effectiveness could be dependent on mounting both an antibody and cell-mediated immune response (214).

Although cell mediated immunity has not been as well-assessed in mumps infection, a lymphoproliferative response was induced in infants vaccinated at 6, 9, or 12 months of age was induced (231) with antigen-specific T-cells reported to appear within 1 month of infection (183). Lymphoproliferative responses to measles and mumps vaccine viruses were shown to persist in two thirds of the population at least 6 years after immunization (232), with T- and B-cell immunity persisting for 10 years post-immunization (202).

Low levels of mumps-specific memory B-cells have also been documented suggesting that mumps infection or vaccination may not generate a robust B-cell memory (136, 233). Two principal mechanisms for maintaining long-term humoral immunity have been proposed and reviewed by Amanna et al. (218): associations between memory B-cell levels and antibody may reflect an epiphenomenon in which serum antibody levels and memory B-cells are equally stable but independently maintained. If memory B-cells and plasma cells are independently regulated, then multiple re-exposures to antigens may cause divergence between memory B-cell levels and antibody levels (218). Antigens with the highest rates of boosting through vaccination or latent viral infection coincidentally showed the weakest association between memory B-cell titers and antibody titers (234).

Although the role and efficacy of T-cell immunity to mumps infection is unclear, there is a possibility that certain MuV strains may be capable of escaping vaccine induced T-cell responses, which may not be considered of significance until B-cell waning immunity comes into play (198). In individuals who did not respond to vaccination (i.e., had a ≤2-fold of mumps antibody titers 30 days post-vaccination), several genes including those implicated in antigen presenting, processing, T-cell response and function showed significantly increased expression, with MHC Class II HLA-DRB3 and HLA-DRA, and CD86 induced when compared to responders 1 day post-MMR vaccination. This may indicate that the stimulation of a rapid adaptive immune response limits antigenic presentation and hence prevent the differentiation of memory B-cells to antibody-producing plasma cells (154).

Differences in predicted B-cell and T-cell epitopes between JL5 vaccine strain and other vaccine strains may also be implicated in the outbreaks witnessed (235). Although, it has also been shown that natural mumps infection or vaccination do not always induce both cellular and humoral immunity. de Wit et al. (199, 236, 237) has shown the presence of Th1-type CD4^+^ T-cells recognizing a MuV epitope in a HLR-DR restricted manner. In addition, the response of IFN-γ and TNF producing CD8^+^ T-cells specific to MuV epitopes are lower in vaccinated individuals when compared to individuals who were naturally infected (199, 213, 236–238). Utilizing current knowledge and new technologies may help define a better surrogate correlate of protection and potentially determine a cut-off between the immunity of a vaccinated individual and a secondary mumps infection. This may potentially move the diagnostic preference from serological tests to more comprehensive functional assays.

### Why Vaccinate If You Cannot Define Protection?

Despite the large resurgence of mumps outbreaks, there is insurmountable evidence highlighting the benefit of the mumps vaccine (Table 2). Routine childhood MMR vaccination has resulted in a dramatic decrease in the incidence of mumps cases, and has shifted the peak age-specific attack rates from a young children (manifesting between 5 and 15 years) to one that affects young adults, in particular those who have close interaction with other young adults (18–24 years) (6, 110). Additionally, clinical manifestations and severity of disease in vaccinated vs. unvaccinated individuals differ (129, 248). Although MuV can be clinically asymptomatic in about 15–30% of those who become infected, the vaccine against mumps confers protection in a dose response manner; unvaccinated individuals saw an attack rate of 31.8–42.9%, whilst one dose and two doses of the JL vaccine were 4–13.6% and 2.2–3.6%, respectively (135, 219, 246).

**Table 2:** Differences between Mumps vaccinated and unvaccinated persons.

	**Vaccinated**	**Not vaccinated**
Symptoms (7, 101, 239, 240)	Milder	Severe
Transmission (197, 241, 242)	Low	High
Mumps viral load and replication (243–245)	Low	High
Mumps isolation rates (135, 239)	Low	High
Duration of viral shedding (244)	Shorter	Lasts Longer
Asymptomatic infection (135, 246, 247)	66%	15–40%

*Despite evidence of mumps infection in a vaccinated population, there is evidence to suggest a less severe clinical manifestation of the viral infection*.

Based on the reduction seen upon the introduction of a mumps vaccine, it has been proposed that MMR vaccination also prevents the transmission of the virus. There is limited knowledge regarding the shedding and transmission of MuV, but it is thought that close contact and transmission of a certain viral load may induce clinical symptoms (243, 246, 249). Modeling data suggests that infectious MuV shedding decreases rapidly after the onset of symptoms, however 8–15% are patients are thought to still be virally shedding 5 days after the onset of symptoms (244). This could be the reason why the transmission of MuV can be exacerbated by close social situations within a heterogeneously vaccinated population. Outbreaks generally occur in situations of intense contact such as college dormitories, boarding schools, and youth summer camps (191), with up to a third reporting some contact with a mumps case (105).

Evidence of lower levels of viral replication also suggests a clinical benefit of the vaccine (243, 244). Viral load and presence of the mumps vaccine genome in areas of viral replication was lower in vaccinated individuals vs. unvaccinated individuals (243). In addition, patients who contracted mumps but had two doses of MMR have been shown to shed less MuV in their urine, with fewer experiencing bilateral parotitis or orchitis than unvaccinated individuals (239), This suggests that immunity induced by MMR vaccination limits virus transmission and complications (241, 242).

It should be noted also that individuals who received two doses of MMR, and had a positive correlation between viremia, salivary viral loads and systematic clinical mumps infection may have an increased risk of transmitting virus. These individuals also lacked mature functional responses, with low neutralizing antibody titers and avidity indexes (239).

Overall, evidence demonstrates a clinical advantage to receiving a mumps vaccine (Table 2). Currently no global consensus exists for the measurement of mumps antibodies, mumps avidity or neutralizing titers that correlate to vaccine response and protection in healthy individuals. If a biomarker is discovered, it could be utilized as an international diagnostic reference standard to allow global harmonization and evaluation of the relative effectiveness of the different vaccination programs worldwide. Such an attempt was conducted by Andrews et al. (250), who reported on the European Sero-Epidemiology Network project which was established to harmonize the seroepidemiology of five vaccine preventable infections including measles, mumps, and rubella in eight European countries. The study concluded that the development of an international standard for mumps would help in the standardization and comparability of mumps antibodies in the different enzyme immunoassays used in laboratories. However, to date, no international reference standard for mumps has been established.

### Can Improvements to Vaccines Be Made?

In response to infection, the human immune system launches a series of immunological responses with the goal of controlling or eliminating the pathogen. If the pathogen circumvents the frontline defense of the innate immune system, an adaptive immune response specific for the pathogen will become activated to respond, with the intention to generate humoral- and cell-mediated immunity. Humoral immunity, represented by antibodies secreted by B-cells are not effective against pathogens that invade host cells. Therefore, cell-mediated immunity instructed by the innate immune system are additionally necessary and consist of B-cells and T-cells. The unique compositions of the B-cell receptor and T-cell receptors specific for the invading pathogen proliferate and gain effector functions based on the antigen fragments presented on antigen presenting cell by MHC class II molecules. The activated Th-cell produces cytokines, resulting in the activation of macrophages (Th1 help), B-cells (Th2 help, called plasma cells), or cytotoxic T-cells. While most plasma cells, produce and secrete large amounts of antibodies, some differentiate into memory cells [reviewed in (251, 252)].

Vaccination aims to stimulate the host immunological process and formation of cell-mediated immunological memory via the use of live-attenuated or of inactivated/subunit vaccine components to promote a cell-mediated immune response. Extensive knowledge gaps significantly hinder improvements to the mumps vaccine and prospects for mumps eradication and maintaining proficient population immunity (3, 122, 187). Few studies have collected data that examines different aspects of mumps immunity and are limited in their predictive value for future outbreaks (253). For example, the importance of T and B-cell responses in protective mumps immunity and how memory/plasma cell numbers are homeostatically maintained post-infection or vaccination is relatively unknown (252). It should be acknowledged that the mechanism of protection of infection may not be the same mechanism of recovery from infection, which may make the identification of a common correlate of protection and recovery difficult (203). Therefore, if a correlate or surrogate correlate is unobtainable to define an individual's protection to mumps, should we re-consider and re-focus efforts on optimizing the vaccine using available historical clinical and trial data?

### Administration

It has been suggested that wild-type infection could confer a “better quality,” broader and prolonged immuno-activation than vaccine-induced immunity. This is reflected in mean neutralizing antibody titers detected post-mumps vaccination, which were over five times lower than those detected following wild type infection. Similarly, hemagglutination-inhibiting titers after natural disease were 1:9 compared to 1:5 post-vaccination (214, 218, 219).

The use of a live-attenuated virus vaccine is intended to mimic immunological reactions and responses between the host and wild type virus (254). The current live-attenuated MMR vaccine is intramuscularly injected, a route that significantly differs from the natural infection mode of transmission. However, emphasized by differing immunological kinetics between immunized and naturally infected individuals when subjected to wild type pathogens, injectable vaccines are considered not to be the best inducer of antigen-specific mucosal immune responses for mucosal pathogens, especially if the mode of administration is not the natural route (the respiratory tract) (255, 256). Improvements on a broader range of antigen delivery systems will improve vaccination strategies and potentially prolong the effect of a vaccination by producing a localized immunological response in the relevant tissues (257, 258).

Mucosal vaccines such as intra-nasal vaccination have advantages over traditional injectable vaccines as they can induce an effective, more robust immune response without any physical discomfort and more closely replicate the natural route of infection for mumps (255, 259). B-cells induced by the mucosal response are also capable of secreting IgA class of antibodies in the lumen, where the interaction and neutralization of specific antigens form IgA-antigen complexes are easily able to be entrapped in the mucus and eliminated by cilial epithelial cells (259). Activated mucosal lymphocytes can also reach other mucosal sites via the lymphatic system and have the capability to transfer immunity (260).

Such an example is the intranasal immunization of inactivated influenza. With a 70–90% similar efficacy between the injectable and intranasal influenza in healthy individuals this intranasal vaccine can elicit the secretion of haemagglutinin and neuraminidase specific IgA antibodies in the upper respiratory tract, and corresponding IgG antibodies (258). Live, cold adapted attenuated nasal influenza vaccine has been routinely used in Russia for over 50 years (261). Other liquid live-attenuated intranasal vaccines are available; “Nasovac®” in India, and “FluMist®” in the US, UK and New Zealand (258, 259, 262).

### Development of Improved Vaccines

Inactivated vaccines consisting of heat/chemical or live-attenuating monovalent or multivalent pathogens in animals/cell lines were developed to protect against disease causing microorganisms (263). Less emphasis was placed on understanding the mechanisms related to conferring immunological memory; the focus lay on the availability, mass production and administration of the vaccine to introduce herd immunity into populations (264).

Currently, the least expensive and time effective method to licensure is the comparison of serologic responses of the new vaccine to an existing licensed vaccine, which can lead to a bias on the development of novel vaccines (222). This methodology also does not account for the fact that each vaccine developed elicits its own immunological signature and may need to be considered on an individual basis (265).

Raymond et al. (266) has suggested that embryonated chicken egg-based vaccines may induce antibodies that are more preferential to egg adapted strains better than wild type virus. Amino acid substitutions/differences in key antigenic targets due to the passage of the growing virus within this environment may optimize the growth of the virus, but could lead to differences over time that could affect the immunogenicity or potency of the vaccine (172, 222, 267). The JL vaccine contains two isolates of the JL Strain (JL2 and JL5) and whilst no immunological differences have been documented, JL2 grows to higher titers than JL5 in embryonic eggs and also demonstrates significant sequence variability (94, 268). Zost et al. (269) also demonstrated that an egg selected mutation within a glycosylation site in the 2016–2017 influenza vaccine strain led to the production of poorer neutralizing antibodies to the vaccine strain compared to wild type influenza virus.

Vaccine RIT 4385 strain derived from one of the two distinct virus subtypes of the JL vaccine (JL5) showed comparable seroconversion rates despite inducing a significantly lower geometric mean antibody titer when compared to recipients of the JL vaccine, but does not have any longitudinal trials investigating its efficacy, even though there are populations who are currently receiving it (101, 270).

The significant time gap between pathogen emergence and vaccine licensure, could potentially lead to antigenic drift. There is potential that modern biotechnologies could be utilized to design novel vaccine platforms (251, 271, 272). Clinically derived recombinant MuV lacking the expression of the immunomodulatory V or SH protein are currently being investigated (273). In China, a vaccine consisting of the prevalent wildtype virus genotype (F) has recently been produced and is currently undergoing trials (269).

In addition, despite being extremely pleomorphic, utilizing MHC epitopes as potential B-cell and T-cell vaccine candidates are also being investigated (81, 274, 275). Vaccine design has involved the utilization and templating of epitopes that previously induced a B-or T-cell response during natural disease that are considered to be immunogenic enough to induce similar responses if administered in a vaccine. However, the appropriate B-cell and T-cell epitope/peptide candidates to induce a protective immunological response can be difficult to correctly identify and synthesize, as it may differ to the immunodominant epitope and host presentation of that antigen (251, 276). Prediction of MHC-peptide binding and cleavage has demonstrated mismatches in both vaccine T-cell and B-cell epitopes in vaccinated individuals highlighting small number of distinguishing amino acid changes of the JL5 major strain (235). The importance of understanding T-and B-cell responses and how antigen-specific memory cells numbers are homeostatically maintained post-infection is crucial to understand to ensure successful vaccine development (252, 277).

Since the 1990's, significant progress has also been made in developing flexible, amplifiable, scalable, inexpensive, and cold-chain free RNA vaccines, such as synthetic mRNA molecules encoding only the antigen of interest and self-amplifying RNA (sa-RNA) (264). Such examples include an experimental mRNA vaccine candidate (mRNA-1273) which encodes a stable form of the SARS-CoV-2 spike protein and has been accepted as a trial candidate for clinical trials in healthy male and female individuals (278, 279). In addition, sa-RNA viruses as gene delivery and vaccine vectors have also demonstrated therapeutic efficacy in a number of preclinical studies. In the context of influenza, sa-RNA vaccines have shown comparable results of protection at lower doses than mRNA vaccines (272, 280, 281).

Exponential developments in the “OMIC” area has enabled further vaccine development and understanding of the immunological response and challenges surrounding this area (282). Systems vaccinology, which includes immunoformatics, DNA/RNAseq, microarrays, mass spectrometry proteomics, transcriptomics, and metabolomics have all shown huge potential in elucidating differences in vaccine strains, vaccine growth and individual response in depth and on an epigenetic level allowing the identification of new vaccine antigens with increased speed and sensitivity (235, 263, 283–285).

Adjuvants, a group of biological and chemical compounds could also be considered to enhance and improve the longevity of the immune response of a vaccine such as the MMR. Adjuvants have been successful in significantly reducing overall antigen dose in vaccine formulations as well as alter and broaden the host response through epitope spreading and qualitatively shaping the effector function of antibodies through subclass selection (173, 286).

The re-purposing of live-attenuated vaccines as TIbV are also being investigated. Trained Immunity based Vaccines (TIbV) elicit heterologous protective effects by inducing a broader, lasting priming of innate immune cells, in addition to the intended specific immunological response and memory of conventional vaccines [reviewed in (287)]. MMR and BCG vaccines have been considered as potential TIbV in the context of the current coronavirus disease 2019 (COVID-19) pandemic (288), however further research is needed.

### Potency of Virus

The mumps component of a vaccine is an unpurified product whose potency is measured through a biological assay for the substance rather than through evaluation of integrity of physical form (quantitative PCR after cell culture) (289). A monovalent mumps vaccine lot is used to characterize the performance of the mumps potency assay with international reference standards. Degradation products are neither identified nor quantified (290). Currently, the minimum potency of the mumps vaccine used varies between brands used [summarized by Su et al. (107)] (291). However, this potency measurement differs to other MMR vaccines strains previously used [reviewed in (10)]. In addition, the maximum required potency is not usually specified. Atrasheuskaya et al. (172) demonstrated that the four out of 14 lots of vaccine associated with six cases of viral transmission post-vaccination to previously vaccinated contacts were in fact twice as potent as the lots that were not associated with viral transmission post-vaccination (172, 292). This may impact the use and efficacy of specific vaccines. Due to their neurovirulence and increased incidence of aseptic meningitis and mumps cases, the Urabe Am 9 and Rubini mumps vaccine strains were discontinued in many countries (87, 293, 294).

Comparing alternative culturing technologies and defining a viral potency range for vaccines could help reduce variability within the MMR vaccine (292). Ensuring the use of a reference sample that had similar replication rate and composition as the virus to be tested will allow accurate determination of the quantity of virus present per lot of vaccine. Investigating novel vaccine candidates shown to induce a similar quantity but qualitatively different antibodies will help segregate and reveal potential correlates of protection (209). Incorporating more modern technologies such as microarray technology or antibody pattern/profiling (rather than single antibody measures) to investigate biomarkers of neutralizing antibody response and/or correlates of protective immunity, in addition to incorporating what has been accomplished in Finland will allow further understanding of mumps immunity (123, 124, 173, 195, 196, 295).

### Are the Current Perceptions of What Is Expected of a Vaccine Skewing the Overall Benefits It Elicits?

The efficacy of a vaccine is defined by disease prevention (sterile immunity, establishment of primary infection and shedding of mature virus particle), or complications associated with infection (orchitis, neurological issues etc.) (203). Despite the well-documented success of the global immunization programs demonstrating how vaccines significantly attenuate disease and onward transmission of infection, they are rarely totally efficacious (demonstrated in pre-licensure clinical trials) or effective (determined by practical use) (99, 173, 296).

Therefore, does “immunity” refer to sterile immunity or solely to protection from symptomatic infection? What defines an effective vaccine, or what constitutes vaccine failure? Does the medical profession and the “pro-vaccine” message contribute to the public skepticism regarding immunization? Is it time to shift the medical and public perception paradigm from “protection of infection following vaccination” to “protection from serious clinical mumps manifestation”?

The lack of definition leads to misinterpretation by health professionals and media of what is truly occurring. Such an example is currently observed with influenza; individuals who have recently being vaccinated against influenza and subsequently become infected with influenza, assume that the vaccine has “failed” even though there is a reduction in symptoms.

The current assertion that vaccines “protect against” or “eliminate” the risk of infection may contribute to the misperception about what level of protection a vaccine actually provides (vaccination efficacy) perpetuated by the witnessing of visible clinical disease and outbreaks despite vaccination (116, 297, 298). Therefore, definition and consensus of what is termed a true “vaccine failure” is required to inform both the clinical and public perception of what the function of a vaccine is. Deciding what the clinical endpoint of a vaccine is i.e., infection with mild clinical symptoms vs. natural infection/disease with its associated complications and assessing the impact of the vaccine in a heterogeneously vaccinated population will allow a better consensus of what is required.

A paradigm shift in what is considered to be a good vaccine i.e. one that provides protection against serious clinical sequalae, in addition to identifying a reliable laboratory marker for this protection is required (203). By focusing on, and acknowledging that vaccines may not prevent infection but will attenuate the clinical complications/consequences that arise from infection in addition to reducing onward transmission will provide a more realistic view of the benefits of vaccination (297). Immunity is therefore beneficial but does not necessarily mean protection.

## Discussion

If we can decide whether the end point of a vaccine is either the prevention of infection or protection against serious sequalae of infection, its efficacy and impact can be determined and will have enormous implications on how vaccine failure can be studied, quantified and interpreted. This teasing out of the immunological response to MuV will ultimately provide potential correlates with robust predictive power, suggest directions for further vaccine improvement, and enable the discovery of potential biomarkers to help create a more efficient diagnostic assay that can discern between different infectious diseases and vaccination vs. disease status. The identification and incorporation of a correlate into diagnostic protocols which can be widely accessible may potentially allow global harmonization of criteria defining immunological protection against mumps.

The medical and scientific field needs to inform the public more accurately about what a good vaccine consists of, which may result in a more positive attitude toward vaccines. In the majority of individuals, a vaccine can prevent serious clinical sequalae and associated complications following wild type infections, but also significantly reduce onwards transmission in particular to the cohorts who are not vaccinated due to a contraindication to vaccination. This is the positive and realistic view of vaccination which should be presented rather than the current flawed message of “get the vaccine and be protected from infection.” The public deserves, and will appreciate, a more accurate and informed message.

## Author Contributions

AC, JC, and JH contributed to the conception and design of the review. AC wrote the first draft of the manuscript. JC, TL, and JH contributed to manuscript revision. All authors have read and approved the submitted version.

## Conflict of Interest

The authors declare that the research was conducted in the absence of any commercial or financial relationships that could be construed as a potential conflict of interest.
